# Mental Health and Cognitive Outcomes in Patients Six Months After Testing Positive Compared with Matched Patients Testing Negative for COVID-19 in a Non-Hospitalized Sample: A Matched Retrospective Cohort Study

**DOI:** 10.3390/ijerph22081249

**Published:** 2025-08-09

**Authors:** Brooklyn Ward, Nicole E. Edgar, Chloe Ahluwalia, Eileen Huang, Daniel Corsi, D. William Cameron, Ian Colman, Mark Kaluzienski, Heather Orpana, Sarah E. MacLean, Simon Hatcher

**Affiliations:** 1 Neuroscience Program, Ottawa Hospital Research Institute, 406-1919 Riverside Drive, Ottawa, ON K1H 1A2, Canada; broward@ohri.ca (B.W.); nedgar@ohri.ca (N.E.E.); cahlu009@uottawa.ca (C.A.); eihuang@toh.ca (E.H.); samaclean@ohri.ca (S.E.M.); 2 Department of Obstetrics and Gynecology, University of Ottawa, 501 Smyth Road, Ottawa, ON K1H 8L6, Canada; dcorsi@ohri.ca; 3 Inflammation & Chronic Disease, Ottawa Hospital Research Institute, 501 Smyth Road, Ottawa, ON K1H 8L6, Canada; bcameron@ohri.ca; 4 Department of Medicine, University of Ottawa, 1145 Carling Avenue, Ottawa, ON K1Z 7K4, Canada; 5 School of Epidemiology & Public Health, University of Ottawa, 600 Peter Morand Crescent, Ottawa, ON K1G 5Z3, Canada; icolman@uottawa.ca; 6 Department of Mental Health, The Ottawa Hospital, 501 Smyth Road, Ottawa, ON K1H 8L6, Canada; 7 Centre for Surveillance and Applied Research, Public Health Agency of Canada, 130 Colonnade Road South, Nepean, ON K2E 1B6, Canada; heather.orpana@phac-aspc.gc.ca; 8 Department of Psychiatry, University of Ottawa, 1145 Carling Avenue, Ottawa, ON K1Z 7K4, Canada

**Keywords:** COVID-19, SARS-CoV-2, coronavirus, mental health, cognitive symptoms, cohort study

## Abstract

We aimed to determine the mental health and cognitive outcomes at six months in people who had not been hospitalized with COVID-19 and who had tested positive or negative for COVID-19 in Eastern Ontario, Canada. Participants were matched 1:1 six months following their COVID-19 polymerase chain reaction test. X2, *t*-test, and Mann–Whitney U tests were conducted to compare self-report and observer-rated mental health and cognitive outcomes between the two groups. We also conducted an age and gender-adjusted logistic regression analysis to explore risk factors associated with depression, anxiety, and cognitive impairment among those who had tested positive for COVID-19. A total of 324 participants were enrolled (*n* = 162 per arm). Overall, 40.7% of those in the COVID-positive group were men, with an average age of 37.9 (SD 13.2) years. In the COVID-negative group, 41.4% were men, with an average age of 36.7 (SD 12.8). There were no statistically significant differences in mental health outcomes between the groups. On cognitive testing, while 21% of the COVID-positive participants and 14% of the COVID-negative participants had scores indicating significant cognitive impairment, the difference between groups was not significant, though this warrants further investigation in future research. In non-hospitalized patients who have tested positive for COVID-19, there is no evidence of an increase in mental health disorders compared to people who tested negative. Any increases in mental health disorders during the pandemic may be the effect of social changes rather than an effect of the virus itself. The exception may be the cognitive changes in those who tested positive.

## 1. Introduction

In March 2020, the World Health Organization (WHO) declared the novel coronavirus (COVID-19) a global pandemic [1]. Previous pandemics have been associated with widespread recognition of their mental health consequences. In 1892, at the time of the “Russian Flu”, William Osler wrote this about influenza: “The depression of spirits following this disease is one of its most unpleasant and obstinate features” [2]. A systematic review and meta-analysis of the mental health consequences of severe coronavirus infection found 40 studies (35 in Severe Acute Respiratory Syndrome [SARS] and 5 in Middle East Respiratory Syndrome [MERS]) that reported ongoing psychiatric symptoms after the initial infection had resolved [3]. The meta-analysis found the point prevalence of mental disorders after the acute illness was 32.2% for Post-Traumatic Stress Disorder (PTSD) (95% CI 23.7–42.0%), 14.9% for depressive disorders (12.1–18.2), and 14.8% for anxiety disorders (11.1–19.4). Only one study examined cognitive function, which found that 15/45 patients (33%) reported a dysexecutive syndrome consisting of inattention, disorientation, or poorly organized movements in response to command [4]. About half of the studies were rated as low quality, with the main weaknesses being limited assessment of psychiatric disorders prior to infection and the absence of control groups [3].

Currently, much of the research on the psychiatric and cognitive outcomes of COVID-19 infection has been in patients who have been hospitalized, despite more than 95% of cases never being admitted to hospital [5,6]. Some literature has explored the outcomes in non-hospitalized samples, though few studies have been conducted examining populations within Canada. A study published by Kang et al. (2024) used population-based data to show that psychological distress is negatively associated with cognitive performance, though this study primarily focused on older adults [7]. A systematic review of the neuropsychological and psychiatric sequelae after COVID-19 found 33 studies, with only 5 including non-hospitalized participants and 3 with any control group [8]. Individuals who were not hospitalized with COVID-19 had a prevalence of reported depression of 15.0 to 68.5%, anxiety of 14.0 to 55.2%, and PTSD of 7%. These figures were similar to those who required inpatient hospitalization. Risk factors included female sex, higher perceived stigma related to COVID-19, having a family member who tested positive for COVID-19, social isolation, medical morbidity, past psychiatric history, identifying as LGBQT+ and low socio-economic status (SES) [9,10]. Cognitive impairment was recorded in 15% to 40% of participants. The wide range in prevalence was attributed to the heterogeneity of the patient samples as well as different assessment methods and timeframes. A subsequent systematic review focused only on depression after COVID-19 and included eight studies, only two of which included a control group [11]. Clinically significant depression was found in 3 to 12% of participants. Nersesjan et al. (2022) found no difference in mental health outcomes at six months in hospitalized COVID-19 patients compared to controls hospitalized for other reasons [12]. Cognitive outcomes were significantly worse in the COVID-19 population (absolute difference was small). A large retrospective cohort study using electronic health records found six-month incidence rates in non-hospitalized COVID-19 patients of 13% for mood disorders, 17.5% for anxiety disorders, and 5.9% for substance use [13]. Cognitive outcomes were not assessed in this study, and there was no general population control group. Xie et al. (2022) used health service records to examine a cohort of US veterans who had evidence of COVID-19 infection and two control groups [14]. Of the COVID-19 cohort, 14% had been admitted to the hospital. They found that veterans infected with COVID-19 had higher rates of mental disorders than the control groups after a year. The population in this study was 90% male and mostly white, which limits its generalizability, and cognitive function was not systematically assessed. In addition, health service record studies are confounded by health-seeking behaviour and access to services, which may differ among those diagnosed with or without COVID-19; the unknown completeness of records; no validation of diagnosis; and little information on socioeconomic risk factors. A systematic review of cognitive impairment associated with COVID-19 found two studies in non-hospitalized patients with follow-up after 12 weeks, neither of which had a comparison group [15]. In studies that reported follow-up after 12 weeks, cognitive impairment was detected in 21 to 65% of people. A systematic review examining attention and memory after COVID-19, measured by neuropsychological tests, found that memory and attention may be affected as a result of infection and suggested controlled studies as a potential solution to better understand the effects of COVID-19 [16].

The current literature primarily focuses on hospitalized cohorts and usually does not include comparison groups of those who tested negative for COVID-19. Studies published more recently that examine non-hospitalized samples focus more heavily on cognition and memory, with no reporting on mental health outcomes, and do not include a control group [17]. There have been few studies examining the outcomes of non-hospitalized Canadian populations. Not including control groups of uninfected individuals means that it is impossible to separate the effects of the virus from the stresses associated with the pandemic, such as gathering restrictions, workplace and school closures, disruption to services, and economic impacts. The primary aim of this study was to determine the mental health and cognitive outcomes at six months in people who had not been hospitalized with COVID-19 and who had tested positive and negative for COVID-19 in Eastern Ontario, Canada. We also wanted to assess any clinical, social, or demographic predictors of mental health and cognitive outcomes in these populations. The results of this study are reported according to the Strengthening the Reporting of Observational Studies in Epidemiology (STROBE) guidelines [18].

## 2. Materials and Methods

### 2.1. Study Design and Participants

This study is a matched retrospective cohort study of non-hospitalized participants who tested positive or negative for COVID-19 at The Ottawa Hospital (Ottawa, Canada) between July 2020 and January 2021. During this time, The Ottawa Hospital operated a large community COVID-19 testing facility in the city. This study reports on outcomes at six months post-PCR testing.

All participants were identified by The Ottawa Hospital Performance Measurement team, who keep a comprehensive list of individuals who have completed a COVID-19 polymerase chain reaction (PCR) test and who consented to be contacted for research purposes. All PCR tests were completed in the context of clinical care and not as part of the current research study. At the time this study was conducted, the primary method for detecting COVID-19 in the general population in Ontario was through PCR testing, and this continues to be the gold standard for testing due to its sensitivity and accuracy [19]. COVID-19 test-positive patients were identified from the list of people tested for COVID at The Ottawa Hospital based on the following criteria: (1) age > 18; (2) positive PCR test within the previous six months; and (3) willingness to consent to the use of email to communicate with the study team. Exclusion criteria included hospitalization for COVID-19. COVID-positive patients were included in the study regardless of whether they were symptomatic at the time of their initial PCR test. Potential COVID test-negative participants were selected from the list of people tested for COVID at The Ottawa Hospital based on the following criteria: (1) age > 18; (2) negative PCR test; (3) willingness to consent to the use of email to communicate with the study team; and (4) matching one-to-one with COVID-positive patients by sex, age difference < 5 years, and PCR testing date within 30 days of the matched test-positive case.

Given that this study relies on patient-reported and clinician-rated outcomes not routinely collected as part of clinical care, participants from both the COVID-positive and matched COVID-negative groups were approached to complete informed consent procedures. If the matched COVID-negative patient declined to participate, another suitable COVID-negative patient was identified and approached. To ensure that COVID-negative participants had not contracted COVID-19 between their initial negative PCR test and their one study visit (six months post initial test), their negative status was re-assessed by study staff at enrollment by asking about their symptoms, positive rapid antigen test (RAT), or a subsequent PCR test. While RATs are not as accurate as PCR testing [20], the likelihood of obtaining a false positive result is extremely low (0.05%) [21]. As such, it is unlikely that patients were mistakenly excluded from the COVID-19-negative group based on the results of their RAT. To account for the possibility of false negatives on a RAT, symptom inquiry was guided by public health criteria at the time, which included fever/chills, cough, shortness of breath, loss or reduction in sense of smell or taste; or if they had two or more of the following: sore throat, headache, runny nose/nasal congestion, extreme fatigue, muscle/joint pain, or gastrointestinal symptoms.

Ethics approval was obtained from the Ottawa Health Sciences Network Research Ethics Board (OHSN-REB; Approval: 20200713). Eligible participants provided verbal consent and confirmed their consent by electronic acknowledgement through the electronic data capturing system (EDCS), prior to the completion of the self-report questionnaires.

### 2.2. Study Visit

All study visits were conducted remotely by research staff at the Ottawa Hospital Research Institute. The single study visit occurred six months post-COVID-19 PCR test result and included a series of self-reports and observer-rated assessments to evaluate mental health and cognition. Upon study enrollment, participants completed a COVID-19 medical history form, which assessed their current COVID-19 status, and among those who had tested positive for COVID-19, it assessed when symptoms first began as well as their severity. Prior to the scheduled visit, participants completed self-report questionnaires to assess depression, anxiety, substance use, PTSD, and psychosis. If the participants met the definition of a case on the self-report scales, indicating the possible presence of a mental health disorder, a trained research assistant administered a complete diagnostic interview as appropriate. This is a standard and reliable approach used in comparative mental health research [22]. All participants completed the Mini-Addenbrooke’s Cognitive Assessment (M-ACE) [23], which was administered virtually via video call with a delegated and trained research staff member, where audio and video were on for the duration of the assessment. For the clock drawing portion of this assessment, participants were observed drawing this on video and were asked to show the research staff their clock once it was complete.

### 2.3. Outcomes

An overview of outcome measures and assessment tools administered can be found in Table 1. More detailed information about each assessment, including its psychometric properties, can be found in Supplemental Table S1. The primary outcomes were the proportions of cases of depression, anxiety, PTSD, substance use, cognitive impairment, and low quality of life. Depression was measured with the Patient Health Questionnaire 9-item (PHQ-9) [24,25] and the subsequent Montgomery–Åsberg Depression Rating Scale (MADRS) [26,27] with scores of 10 or higher and 9 or higher, respectively, indicating depression. Anxiety was measured by the Generalized Anxiety Disorder 7-item (GAD-7) [28], followed by the Hamilton Rating Scale for Anxiety (HAM-A) [29] with scores of 10 or higher and 8 or higher, respectively, indicating anxiety. PTSD was measured with the Primary Care PTSD Screen for DSM-5 (PC-PTSD-5) [30] with a score of 3 indicating further screening with the Clinician Administered PTSD Scale for DSM-5 (CAPS-5) [31]. Alcohol and substance use were evaluated with the Alcohol Use Disorders Identification Test (AUDIT) [32], the Drug Abuse Screening Test (DAST-10) [33], and followed by the Alcohol, Smoking, and Substance Interview Screening Test (ASSIST) [34] if potentially hazardous behaviours were identified. Potential psychosis was evaluated using the Psychosis Screening Questionnaire [35] and subsequent Modified Psychotic Symptom Rating Scales (PSYRATS)—Beliefs & Voices Hearing Rating Scale [36]. Lastly, cognitive impairment was evaluated using the M-ACE with a score of 25 or below indicating cognitive impairment [37]. Participants in this study were not specifically assessed for long COVID, as at the time this study was conducted, nothing was known about persistent-COVID symptoms.

Secondary outcomes were severity of fatigue [38], sleep disturbance [39], mental well-being [40], loneliness [41], suicidal thoughts and behaviour [42], and change in work status.

Outcome predictors, potential confounders, and effect modifiers were collected and are described in Supplementary Table S2. We collected information on demographics, medical and psychiatric history, COVID-19 symptoms in the cases, adverse childhood event scores [43], current mental health treatment, and social support [44].

### 2.4. Statistical Analysis

Demographic characteristics and health rating scale scores are presented as mean (SD) for continuous variables and expressed as frequencies and percentages for categorical variables. Primary analyses compared COVID-positive with matched COVID-negative participants. To compare differences in mental health and cognitive outcomes between COVID-positive and COVID-negative (matched sample), we conducted conditional Poisson regressions with robust standard errors to account for matching. Where continuous outcome measures are significantly skewed (skewness greater than 1 or less than −1), we also present medians and quartiles for descriptive analyses. Efforts were made to complete the study visit six months after PCR testing, with a median follow-up time of 6.6 months, quartiles 6.4–6.8. In addition, we conducted non-matched sub-analyses within the COVID-19 test-positive population, using age- and sex-adjusted logistic regression analysis to explore risk factors associated with depression, anxiety, and cognitive impairment.

All statistical tests were two-sided, and an alpha of 0.05 was considered statistically significant unless otherwise stated.

### 2.5. Missingness

Baseline and follow-up data were largely complete, although some observations were missing. We used a complete case analysis as missingness was low and did not exceed 8% on any variable. We noted sample sizes for complete cases when missingness was present.

### 2.6. Sample Size

We based the sample size calculation on the point prevalence of PTSD, as this was the most common disorder reported after SARS and MERS infections, at a rate of 32.2% (95% CI 23.7–42.0;) at 33 months [5]. Given that we were examining a non-hospitalized cohort at six months, we assumed a point prevalence of PTSD of 28% among those who tested positive [3]. In Canada, the general population point prevalence of PTSD is about 10% [45]. In the COVID-negative group, we assumed that the rate of PTSD would increase by 50% to 15% as a result of the general stress of the pandemic. To have 80% power to detect a difference of 13% between the positive and negative groups requires 156 people in each group, or a total of 312 people.

## 3. Results

We recruited a total of 162 COVID-19 test-positive participants and 162 matched COVID-19 test-negative participants between 8 January 2021, and 27 April 2022, who had their initial COVID-19 PCR test between July 2020 and March 2021. The current paper reports on one visit that occurred six months post-COVID-19 test. A total of 1492 potentially eligible cases were identified, and 1426 were contacted. Overall, 964 potentially eligible participants did not answer the phone when recruitment attempts were made. Per our institutional ethical guidelines, we did not attempt to contact anyone more than three times. Seven individuals had out-of-date contact information, so we were unable to reach them. Of 284 participants who were not interested in participating, reasons for noninterest included personal time constraints (*n* = 29), mental health or burnout (*n* = 5), distrust in research overall (*n* = 1), language barrier (*n* = 1), and already involved in clinical trials and were asked by their other research study team not to participate in any other research (*n* = 2). The rest of those uninterested (*n* = 246) did not wish to provide a reason. Two individuals were hospitalized for COVID and were ineligible for the study. We enrolled 169 cases, and 162 cases completed the survey, with a response rate of 11.4%.

Further, 2413 matched potentially eligible controls were identified after cases completed the survey, and 1522 were contacted before matched recruitment was complete. Of potentially eligible control participants, 1026 did not answer the phone when recruitment attempts were made. Of 243 who were not interested in participating, reasons for non-interest included personal time constraints (*n* = 36), mental health *(n* = 5), and the rest of those uninterested (*n* = 202) did not wish to provide a reason. Two controls had tested positive at the time of contact and were ineligible, and eight had out-of-date contact information and were unable to be contacted. We enrolled 243 controls, with 162 completing the survey, with a response rate of 10.6%. A flow diagram to further illustrate this process is in the Supplemental File (Figure S1).

The demographic and clinical characteristics of the two groups are provided in Table 2. The mean age of positive participants was 37.9 years (SD 13.2), and 66/162 (40.7%) were male sex, and the negative participants had a mean age of 36.7 years (SD 12.8), with 67/162 (41.4%) being male sex. In both groups, participants were mostly married or in a common-law relationship, working full-time, with a post-secondary education. There were no significant differences between the two groups in age, sex, housing status, education, employment, income, the proportion of health care workers, or adverse childhood event scores. There was a statistically significant difference in the proportion of participants who self-identified as being Black, Indigenous, or a Person of Color (BIPOC), with 24.1% (39/162) identifying as BIPOC in the COVID-19 test-positive group compared to 9.3% in the COVID-19-negative group (15/162) (*p* < 0.001). COVID-19 test-positive participants were also significantly less likely to have a psychiatric history (30/162 [18.6%] vs. 48/162 [29.6%] (*p* < 0.02)), including previous psychiatric or addiction outpatient treatment, a current diagnosis, a history of a suicide attempt, or a family history of mental health problems. In the COVID-19 test-positive participants, 16/162 (10.3%) reported no COVID-19 symptoms at the time of their positive test, 95/162 (73.1%) reported a loss of smell, and 31/162 (26.1%) reported confusion or disorientation. Residual COVID-19 symptoms were present six months post-PCR in 40/162 (25.2%) of the cases.

The mental health and cognitive outcomes at baseline are outlined in Table 3. One COVID-positive participant screened positive for psychosis. There were no statistically significant differences in mental health or cognitive outcomes between the COVID-positive and COVID-negative groups. The COVID-negative group was significantly more likely to report problems with pain or discomfort on the EQ-5D-5L compared to the COVID-positive participants, but there was no significant difference in the Visual Analogue Scale (VAS) score. The COVID-negative population scored significantly higher on the AUDIT, although there was no significant difference between the groups in the proportion of participants with hazardous drinking. On cognitive testing, 31/146 (21.2%) of the COVID-positive participants and 20/145 (13.8%) of the COVID-negative participants scored below the cutoff for likely cognitive impairment.

To account for the significantly higher proportion of COVID-19 test-negative participants with a psychiatric history, we did two further analyses, which are reported in Supplemental Table S3. First, we completed both a standard and logistic regression analysis of continuous outcomes adjusted for age, psychiatric history (a composite of positive psychiatric history, a current psychiatric diagnosis, current psychotherapy or counselling, and a history of a suicide attempt), and health care worker status. This confirmed the significant difference in scores on the AUDIT with no other statistically significant differences. We also compared COVID-test-positive and COVID-test-negative participants without a psychiatric history (Supplemental Table S4). Again, this confirmed the statistically significant difference in AUDIT scores with no other differences significant at the *p* < 0.05 level.

Within the COVID-positive group, we examined which variables were associated with mental health and cognitive outcomes (Table 4). We completed a logistic regression analysis and a standard regression analysis adjusted for age and gender for caseness and scores, respectively, on the PHQ-9, the GAD-7, and the M-ACE. This showed that a previous psychiatric history, household low income (below CAD 50,000 a year), and adverse childhood events were associated with the mental health outcomes but not with cognitive impairment. Low income was associated with cognitive impairment. Identifying as belonging to a BIPOC population was not significantly associated with any mental health or cognitive outcomes.

## 4. Discussion

There have been a wide range of estimates of the impact of COVID-19 infection on mental health and cognitive outcomes. Most of these estimates come from unmatched studies of hospitalized COVID-19 patients [8]. Using a matched non-hospitalized cohort who tested negative for COVID-19, considers the psychological impact of the wider pandemic, such as gathering restrictions, disruption to services, economic impacts, reduced socialization, employment impacts, and uncertainty over the future. This study helps to isolate the specific effect of the COVID-19 virus on mental health outcomes. We found no statistically significant differences in mental health outcomes between the two groups, even after controlling for the higher rate of past psychiatric disorders in the COVID-19-negative group. This finding is consistent with the research by Nersesjan et al. [12], which, like this study, used a COVID-19-negative group matched for age and sex but looked at hospitalized COVID-19 patients. That study found similar rates of mental disorders in both groups at six months after symptom onset. Our estimates of the prevalence of mental health disorders are generally at the lower end of previous figures. This could be the effect of using standardized interviews to confirm diagnoses and not relying on self-report rating scales such as the PHQ-9, which have a high false positive rate [46]. There was a non-significant but higher prevalence of harmful alcohol use in the COVID-19 negative group, although the absolute difference is small. We also found no significant difference in cognitive outcomes, although about 21% of the COVID-19 test-positive group were below the threshold for likely cognitive impairment at 6 months compared to 14% in the test-negative group.

The sample contains a lower percentage of men at 41% compared to 46% who tested positive for COVID-19 in Ottawa, although the ages are similar, with a median age of 35 years in the nearly 75,000 who have tested positive as of June 2022 [6]. The focus on non-hospitalized patients is a strength of this article, as most people (71,901 out of 74,446, 96.5%) who tested positive in Ottawa were not admitted to hospital [6]. The strength of this study is the retrospective design and 1:1 matching with a COVID-negative group. The two groups were tested at the same time, experiencing similar degrees of restrictive measures in Eastern Ontario. This means that people who were COVID-19 negative and those who tested positive were exposed to comparable experiences affecting well-being during this period, apart from testing positive for COVID-19, any associated illness, and the subsequent self-isolation.

This study has several limitations. First, the COVID-19-negative group had a higher rate of reported prior psychiatric disorders compared to the COVID-19 test-positive group. A possible reason for this is that, despite sex and age matching, those with mental health struggles may have self-selected into the study, though we attempted to control for this in the analysis. It is possible that this bias resulted in fewer differences between the COVID-positive and COVID-negative groups. Second, as we matched for age and sex in this study, this may occlude important age- or sex-based differences in experiences of the COVID-19 pandemic; for example, a recent United Nations report highlights that women, girls, and gender-diverse people have been disproportionately impacted by the pandemic [47]. Third, this study only collected data at six months post-PCR test and not at the point of infection. As such, we could not assess differences between the initial test and the six-month time point. This could be a consideration for future study design. Fourth, due to limits and changes to PCR testing throughout the study and unknown proportions of asymptomatic infection, it is possible that some COVID-19-negative participants had experienced an infection. We have attempted to control for this by screening at entry to verify multiple testing methods and experience of common symptoms to exclude potential positive cases from the negative group. Fifth, due to the pandemic, many assessments for this study were completed virtually with participants, which may raise validity concerns. This may have especially impacted the cognitive testing in this study, completed using the M-ACE, as it is possible that participants who performed poorly remotely would have received higher scores had the tool been completed in-person (as is the standardized means of administration). It should also be noted that the M-ACE is a cognitive screening tool and, while psychometric properties are robust, may not be as accurate as a thorough neuropsychological evaluation. As such, any increase in cognitive impairment would need to be confirmed through a complete neuropsychological exam. However, the higher proportion of cognitive impairment in the COVID-positive group, though not statistically significant, warrants further consideration for future research. Additionally, rates of possible cognitive impairment are elevated in the COVID-negative group given the demographics of the sample. It is possible that the M-ACE is over-estimating possible cognitive impairment in the control group, and, if this is the case, it is likely that rates in the COVID-positive group could be elevated as well. However, it is also possible that these rates of possible cognitive impairment are reflective of a prolonged state of acute stress and isolation during the COVID-19 pandemic. This would be consistent with the existing literature on the relationship between chronic stress and cognitive functioning [48,49]. Given that we did not complete comprehensive neuropsychological evaluations in this study, it is not possible to definitively determine the cause of these elevated rates of potential cognitive impairment. This is a fruitful avenue for future research. Sixth, the low response rate in both the COVID-19-positive and -negative groups possibly limits the representativeness of the sample, which may affect the generalizability of these findings and could impact the internal validity between group comparisons. Seventh, it is possible that elevated rates in mental health disorders are reflective of the impact of social distancing and other public measures implemented to curb the spread of COVID-19. Finally, this study may have been underpowered to detect clinically important differences in mental health and cognitive outcomes. However, the greatest difference in proportions of people with mental disorders in favour of the COVID-19 group was 7.5%, which suggests that large differences in the prevalence of mental disorders were unlikely to be missed.

This study has important implications for emergency preparedness for future health crises. Since the declaration of COVID-19 as a public health emergency in March 2020, numerous reports have highlighted that emergency response plans were ill-equipped to manage such a large-scale and severe health crisis, with governments criticized for not implementing lessons learned during both the SARS and H1N1 pandemics [49,50]. This is especially true for recommendations intended to curb the mental health impacts of these crises [51,52]. A 2021 WHO report detailing key insights from the COVID-19 pandemic once again highlighted the importance of early provision of mental health and psychosocial supports in health emergencies, especially for high-risk groups, including children, students, health care workers, and the elderly [52]. This study provides support for these guidelines. The results of this study provide nuance to this recommendation, emphasizing that these supports must not only address the impacts of the virus itself but also the mental health load of emergency health measures, including social distancing, lockdowns, and quarantine, a point further supported by emerging evidence from one study indicating that social and psychological effects of the pandemic may also have biological effects [53]. This is especially relevant given the ongoing burden of long COVID. Recognizing the resilience and vulnerabilities within community settings allows for more targeted interventions and resource allocations, fostering more proactive strategies that extend beyond clinical contexts. Navigating the overlap of infectious disease and mental health, the insights gained from this study may contribute to a foundation for comprehensive planning, ensuring a more adaptable approach to mental well-being in future public health crises.

## 5. Conclusions

In summary, in non-hospitalized patients who have tested positive for COVID-19, there is no evidence of an increase in mental health disorders compared to people who tested negative for COVID-19. One possible hypothesis for this is that increases in mental health disorders during the pandemic may be attributable to social changes rather than an effect of the virus itself; however, future research focused on social changes, such as changes in the availability of outreach services, is needed to further assess this. The exception may be the cognitive changes in those who tested positive for COVID-19, though further research is needed to better understand the changes and potential impact on cognition. The results of this study may help to bolster planning activities in preparation for subsequent pandemic emergencies.

## Figures and Tables

**Table 1:** Overview of outcome measures.

Outcome	Self-Report Measure	Observer-Rated Assessment	Cut-Off Score
Depression	Patient Health Questionnaire 9-item (PHQ-9)	Montgomery–Åsberg Depression Rating Scale (MADRS)	PHQ-9 Score ≥10, MADRS completed
Anxiety	Generalized Anxiety Disorder 7-item Scale (GAD-7)	Hamilton Rating Scale for Anxiety (HAM-A)	GAD-7 Score ≥10, HAMA completed
Post-Traumatic Stress Disorder (PTSD)	Primary care PTSD screen for DSM-5 (PC-PTSD-5)	Clinician Assessment for PTSD for DSM-5	Score >3, a CAPS-5 completed
Alcohol Misuse	Alcohol Use Disorder Identification Test	N/A	A score of ≥8 on AUDIT indicates potential problematic drinking
Substance Misuse	Drug Abuse Screening Test (DAST-10)	Alcohol, Smoking, and Substance Interview Test (ASSIST)	ASSIST completed for DAST-10 score ≥3
Psychosis	Psychosis Screening Questionnaire (PSQ)	Modified Psychotic Symptom Rating Scales (PSYRATS)—Beliefs & Voices Hearing Rating Scale	Positive response to 1 anchor and at least 1 probe on PSQ prompted for PSYRATS.
Cognitive Functioning	N/A	Mini-Addenbrooke’s Cognitive Exam (mini-ACE)	Score of 25 for likely cognitive impairment (non-dementia)
Quality of Life	EQ-5D-5L	N/A	N/A
Fatigue	Fatigue Assessment Scale (FAS)	N/A	A score of 22 or higher indicates fatigue.
Sleep	Pittsburgh Sleep Quality Index (PSQI)	N/A	A dimensional score of 5 or higher indicates poor sleep quality.
Mental Well-being	Warwick–Edinburgh Mental Well-being Scale (WEMWBS)	N/A	A score of 42 or lower is indicative of poor well-being.
Suicidality	Columbia Suicide Severity Rating Scale (C-SSRS)	N/A	N/A
Loneliness	Short Loneliness Scale (SLS)	N/A	N/A
Work Status	Change in work status	N/A	N/A

**Table 2:** Participant characteristics.

	COVID-19 Test-Positive (n = 162)	% or (SD) or [IQR]	COVID-19 Test-Negative (n = 162)	% or (SD) or [IQR]	p-Value
Age, years (median, 25th–75th percentiles)	34 (26–44)	[18]	37 (27–44)	[17]	0.24
Male sex	66	40.7	67	41.4	0.32
LGBTQ+	13	8.0	15	9.3	0.72
Black, Indigenous, or person of colour (BIPOC)	39	24.1	15	9.3	<0.001
Marital status					0.73
Single	61	37.7	53	32.7	
Married or common law	87	53.7	93	57.4	
Separated/Divorced/Widowed	9	5.6	13	8.0	
Other/Prefer not to Say	5	3.1	3	1.9	
Housing					0.98
Own my residence	75	46.6	89	54.9	
Rent my residence	72	44.7	55	34.0	
Couch surfing	6	3.7	8	4.9	
Other	8	5.0	10	6.2	
Education					0.12
Less than High school	1	0.6	0	0	
High school graduate	9	5.6	5	3.1	
Some college/University (not complete)	31	19.1	23	14.2	
College education	22	13.6	28	17.3	
University education	66	40.7	62	38.3	
Postgraduate degree	33	20.4	39	24.1	
Other/Prefer not to say	0	0	5	3.1	
Employment					0.89
Full-time	104	64.2	111	68.5	
Part-time	34	21.0	21	13.0	
Short-term disability	1	0.6	1	0.6	
Long-term disability	1	0.6	2	1.2	
Ontario Disability Support Program	0	0	3	1.9	
Student (full or part-time)	5	3.1	12	7.4	
Caregiver/Homemaker	4	2.5	1	0.6	
Retired	2	1.2	4	2.5	
Unemployed	11	6.8	7	4.3	
Income					0.42
Less than CAD 19,999 per year	13	8	10	6.2	
CAD 20,000–49,999 per year	17	10.5	20	12.3	
CAD 50,000–CAD 79,999 per year	31	19.1	31	19.1	
CAD 80,000–CAD 110,000 per year	21	13	29	17.9	
Over CAD 110,000 per year	57	35.2	63	38.9	
Prefer not to answer	23	14.2	9	5.6	
Health care workers					0.62
Health care worker	31	19.1	34	21.0	
Index visit COVID-19 symptoms					
Asymptomatic	16	10.2			
Loss of smell	95	73.1			
Loss of taste	95	72.0			
Headache	108	81.2			
Loss of speech or movement	5	4.2			
Confusion or disorientation	31	26.1			
Time off work (days, mean, SD)	n = 97	12.4 (14.8)			
Time self-isolating (days, mean, SD)	n = 158	14.1 (4.7)			
Duration of symptoms (weeks, mean, SD)	n = 115	3.2 (4.7)			
Residual COVID-19 symptoms at six months	40	25.2			
Medical history					
BMI (kg/m^2^, mean, SD)	28.8	8.2	28.6	10.0	0.85
Diabetes	10	6.2	4	2.5	0.08
Cardiovascular disorders	17	10.5	9	5.6	0.04
Psychiatric history					
Yes	30	18.6	48	29.6	0.016
Previously received outpatient psychotherapy or counselling	43	26.7	68	42.2	0.003
Previous in-patient psychiatric treatment	3	1.9	6	3.7	0.31
Previous treatment for alcohol or drug use	-	-	8	4.9	
Suicide attempt	17	10.6	27	16.7	0.13
Family mental health history	44	27.3	60	37.0	0.047
Adverse childhood event score (median, 25th–75th percentiles)	1.0 (0,2)	[2]	1.0 (0,2)	[2]	0.79

Data are mean (SD), median (IQR), or n (%). *p*-value is from conditional Poisson regression.

**Table 3:** Mental health and cognitive outcomes at six months.

	COVID-19-Positive (n = 162)	% or (SD) or [IQR]	COVID-19-Negative (n = 162)	% or (SD) or [IQR]	Difference in Proportions (95% Confidence Interval)	*p*-Value
Any psychiatric diagnosis	23	14.2	37	22.8		0.040
Any psychiatric medication	24	14.9	35	21.6		0.10
Currently receiving outpatient psychotherapy or counselling	17	10.6	26	16		0.15
Depression						
PHQ-9						
Median score (IQR)	4.0 (2.0–9.0)		5.0 (2.0–10.0)			0.23
PHQ-9 case 10 or greater	33	20.8	41	25.5		0.27
MADRS (when PHQ-9 is ≥10)						
Mean score	15.9	8.0	16.8	9.5		0.66
MADRS case 9 or greater	25	15.4	29	17.9	−2.2 (−9.5 to 5.1)	0.55
Anxiety						
GAD-7						
Median score (IQR)	3.0	1.0–7.0	4.0	2.0–8.5		0.07
GAD-7 case 10 or greater	26	16.3	38	23.8		0.06
HAMA (where GAD-7 is ≥10)						
Mean score	11.7	5.3	14.3	8.6		0.15
HAMA case 8 or greater	18	11.3	27	16.8	−5.4 (−11.9 to 1.1)	0.11
PTSD						
PC-PTSD-5						
Number of cases with score >3	16	10.1	18	11.2		0.77
CAPS-5 (if PC-PTSD-5 >3)						
CAPS-5 Criteria met	14	8.8	16	9.9	−1.2 (−7.5 to 5.0)	0.70
Suicidal thoughts and behaviour						
C-SSRS						
Any positive response	26	16.1	38	23.5		0.13
Quality of life						
EQ-5D-5L (Any problems)						
Mobility	16	10.1	26	16.1		0.07
Self-care	8	5.1	8	5.0		0.97
Usual activities	36	22.6	46	28.6		0.21
Pain/Discomfort	67	42.2	90	55.9		0.01
Anxiety/Depression	88	55.3	98	60.9		0.22
Median VAS score	80.0	69.0–90.0	78.0	70.0–86.5		0.67
Well-being						
WEMWBS						
Mean score	48.8	9.8	48.7	8.8		0.91
Low Well-being (14–42)	38	24.1	40	25.2		0.59
Average Well-being (43–59)	101	63.9	104	65.4		
High Well-being (60–70)	19	12	15	9.4		
Social support						0.75
Poor (3–8)	22	13.8	27	16.8		
Moderate (9–11)	74	46.5	70	43.5		
Strong (12–14)	63	39.6	64	39.8		
Social isolation						
SLS						
Mean score	5.6	2.3	5.9	2.1		0.18
Drug and alcohol use						
DAST-10						
Median score	1.0	1.0–1.0	1.0	1.0–2.0		0.21
Number of cases scoring ≥3	15	9.4	18	11.2		0.60
AUDIT						
Median score	2.0	1.0–5.0	3.0	2.0–6.0	−1.5 (−2.4 to −0.6)	0.002
Number of cases scoring ≥8	21	13.2	30	18.6		0.19
ASSIST						
Cannabis						
Low to moderate use	27	17.0	32	19.9		0.14
Cocaine						
Low to moderate use	23	14.5	26	16.1		0.29
Fatigue						
FAS						
Median score	20.0	16–27	21.0	17–28		0.45
Number of cases ≥ 22	67	42.1	77	47.8		0.24
Sleep						
PSQI						
Mean score	7.4	3.8	7.0	3.5		0.36
Number scoring >5	100	62.9	97	60.2		0.64
Cognitive Functioning						
Mini-Addenbrooke’s Cognitive Exam	*N* = 146		*N* = 145			
Median score	28.0	26–29	28.0	26.5–29		0.051
Number scoring 25 below	31	21.2	20	13.8	6.8 (−1.0 to 14.7)	0.088

Data are mean (SD), median (IQR), or n (%). *p*-value is from conditional Poisson regression.

**Table 4:** Predictors of mental health and cognitive outcomes within COVID-19-positive participants (logistic and standard regression analyses).

Outcomes	Logistic Regression			Standard Regression		
Outcome: PHQ Depression						
Variable	OR	95% CI	*p*-Value	Coefficient	95% CI	*p*-Value
Psychiatric history	4.94	(1.67, 14.57)	0.0038	3.78	(1.88, 5.68)	0.0001
BIPOC	1.03	(0.30, 3.51)	0.9641	−0.65	(−2.75, 1.45)	0.5402
Low income (less than CAD 50,000)	4.36	(1.38, 13.76)	0.0121	2.63	(0.61, 4.64)	0.0111
ACE Score	1.42	(1.14, 1.77)	0.0019	0.65	(0.24, 1.06)	0.0022
Index visit neurological symptoms *	1.53	(0.33, 7.19)	0.5869	−1.16	(−3.45, 1.12)	0.3160
Outcome: GAD-7 Anxiety						
Variable	OR	95% CI	*p*-Value	Coefficient	95% CI	*p*-Value
Psychiatric history	4.98	(1.40, 17.74)	0.0133	3.19	(1.71, 4.66)	0.0000
BIPOC	0.88	(0.21, 3.64)	0.8631	−0.54	(−2.17, 1.09)	0.5131
Low income (less than CAD 50,000)	4.60	(1.16, 18.30)	0.0301	1.44	(−0.13, 3.01)	0.0714
ACE Score	1.54	(1.20, 1.97)	0.0006	0.51	(0.19, 0.83)	0.0021
Index visit neurological symptoms	0.35	(0.07, 1.70)	0.1936	−1.31	(−3.09, 0.47)	0.1474
Outcome: Mini-ACE						
Variable	OR	95% CI	*p*-Value	Coefficient	95% CI	*p*-Value
Psychiatric history	0.76	(0.27, 2.18)	0.6158	0.32	(−1.08, 1.72)	0.6486
BIPOC	1.57	(0.53, 4.63)	0.4119	−0.74	(−2.33, 0.84)	0.3539
Low income (less than CAD 50,000)	4.43	(1.52, 12.94)	0.0065	−1.18	(−2.67, 0.31)	0.1198
ACE Score	1.10	(0.87, 1.39)	0.4126	−0.29	(−0.61, 0.03)	0.0743
Index visit neurological symptoms	0.68	(0.21, 2.18)	0.5169	0.81	(−0.90, 2.51)	0.3501

Adjusted for all factors in the table, age, and gender. * Composite of Loss of smell OR Loss of taste OR Loss of speech or movement OR Confusion or disorientation. BIPOC: Black, Indigenous or Person of Colour; ACE score: Adverse Childhood Event Score.

## Data Availability

The data presented in this study are available on request from the corresponding author due to privacy reasons.

## Supplementary Materials

The following supporting information can be downloaded at: https://www.mdpi.com/article/10.3390/ijerph22081249/s1, Table S1: Outcome Measures; Table S2: Predictors, potential confounders, and effect modifiers; Table S3: Comparison of mean differences in continuous outcomes and regression-adjusted differences; Table S4: Comparison of mean difference in continuous outcomes and regression-adjusted differences-only in participants without psychiatric diagnoses; Figure S1: Participant flow diagram.

## Abbreviations

The following abbreviations are used in this manuscript:

ASSIST	Alcohol, Smoking, Substance Interview Screening Test
AUDIT	Alcohol Use Disorders Identification Test
BIPOC	Black, Indigenous or a Person of Color
BMI	Body Mass Index
CAPS-5	Clinician Administered Post-Traumatic Stress Disorder Scale for DSM-5
COVID-19	Novel coronavirus
C-SSRS	Columbia Suicide Severity Rating Scale
DAST-10	Drug Abuse Screening Test
ECDS	Electronic Data Capturing System
EQ-5D-5L	EuroQol 5-Dimension 5-Level
FAS	Fatigue Assessment Scale
GAD-7	Generalized Anxiety Disorder 7-Item
HAM-A	Hamilton Rating Scale for Anxiety
IQR	Interquartile Range
M-ACE	Mini-Addenbrooke’s Cognitive Assessment
MADRS	Montgomery–Åsberg Depression Rating Scale
MERS	Middle East Respiratory Syndrome
OHSN-REB	Ottawa Health Science Network Research Ethics Board
PC-PTSD-5	Primary Care Post-Traumatic Stress Disorder Screen for DSM-5
PCR	Polymerase Chain Reaction
PHQ-9	Patient Health Questionnaire 9-Item
PSQ	Psychosis Screening Questionnaire
PSQI	Pittsburgh Sleep Quality Index
PSY-RATS	Modified Psychotic Symptom Rating Scales
PTSD	Post-Traumatic Stress Disorder
RAT	Rapid Antigen Test
SARS	Severe Acute Respiratory Syndrome
SD	Standard Deviation
SES	Socio-Economic Status
SLS	Short Loneliness Scale
STROBE	Strengthening the Reporting of Observational Studies in Epidemiology
VAS	Visual Analogue Scale
WEMWBS	Warwick–Edinburgh Mental Well-being Scale
WHO	World Health Organization
