# Supplementary material for: Mental Health and Cognitive Outcomes in Patients Six Months After Testing Positive Compared with Matched Patients Testing Negative for COVID-19 in a Non-Hospitalized Sample: A Matched Retrospective Cohort Study
Source: Int J Environ Res Public Health. 2025 Aug 9;22(8):1249. doi: 10.3390/ijerph22081249 (PMC12386409; doi:10.3390/ijerph22081249)
Supplement: Supplementary file 1 [file ijerph-22-01249-s001.zip › Table S1 - Outcome Measures.pdf]

Table S1. Outcome Measures

Self-Report Measure	Observer Rated Assessment	Measure Description	Cut-Off Score
Patient Health Questionnaire 9-item (PHQ-9)	Montgomery-Åsberg Depression Rating Scale (MADRS)	The PHQ-9 is a 9-item questionnaire that assesses the severity of depression symptoms experienced within the last two weeks, with total scores ranging from 0 (minimal depression) to 27 (severe depression) (Kroenke et al., 2001).<br><br>The Montgomery-Åsberg Depression Rating Scale (Structured Interview Guide) is a 10-item observer-rated scale to assess the severity of depressive symptoms within the last 7 days [25].	PHQ-9 Score ≥10, MADRS completed
Generalized Anxiety Disorder 7-item Scale<br><br>(GAD-7)	Hamilton Rating Scale for Anxiety (HAM-A)	A 7-item questionnaire that assesses the severity of anxiety symptoms experienced within the last two weeks Spitzer et. al, 2006.<br><br>The Hamilton Rating Scale for Anxiety (HAM-A) is a 14-item semi-structured interview evaluating symptoms of anxiety [28].	GAD-7 Score ≥10, HAMA completed
Primary care PTSD screen for DSM-5 (PC-PTSD-5)	Clinician Assessment for PTSD for DSM-5	A screening tool, which consists of four-items which evaluate the presence of PTSD-related symptoms. Prins et al. (2016) recommend using a cut-off score of three (out of a possible four points) to detect possible PTSD [29].<br><br>The CAPS-5 is a structured interview, administered to assess a traumatic event(s) and associated symptoms of PTSD in the past 30 days [30].	Score >3, a CAPS-5 completed
Alcohol Use Disorder Identification Test	N/A	The AUDIT is a brief 10-item questionnaire that assess alcohol consumption, drinking behavior, adverse reactions, alcohol-related problems. Among those who were known to misuse alcohol, the AUDIT successfully detected an alcohol use disorder 99% of the time [31].<br><br>The AUDIT does not require the use of an observer rated assessment to evaluate problematic drinking behaviour and to reduce participant burden the ASSIST was not completed for alcohol use only. However, when administered, the ASSIST also evaluated alcohol use.	Score of ≥8 on AUDIT indicates potential problematic drinking
Drug Abuse Screening Test (DAST-10)	Alcohol, Smoking and Substance Interview Test (ASSIST)	A 10-item questionnaire that assesses drug abuse within the last 12 months with total possible scores from 0 to 10 [32].<br><br>The ASSIST is a clinical interview collecting information regarding use of tobacco, alcohol, cannabis, cocaine, amphetamine type stimulants, sedatives, hallucinogens, inhalants, opioids, and other drugs [33].	ASSIST completed for DAST-10 score ≥3
Psychosis Screening Questionnaire (PSQ)	Modified Psychotic Symptom Rating Scales (PSYRATS) - Beliefs & Voices Hearing Rating Scale	The Psychosis Screening Questionnaire screens for hypomania, thought interference, delusions of persecution, delusional mood and auditory hallucinations. It consists of seven questions and has been used in large population surveys [34].<br><br>The Psychotic Symptom Rating Scales (PSYRATS) evaluates the dimensions of both auditory hallucinations and delusions associated with psychosis. A positive result on the interview was determined by Principal Investigator review [35].	Positive response to 1 anchor and at least 1 probe on PSQ prompted for PSYRATS.

N/A	Mini-Addenbrooke's Cognitive Exam (mini-ACE)	The Mini-Addenbrooke's Cognitive Exam is a brief (5 minute) cognitive screening test that can be used to screen for mild cognitive impairment and is free to use with a maximum possible score of 30 [36]. While this tool is useful tool for screening for mild cognitive impairment, it is not a thorough neuropsychological assessment.	Score of 25 for probable cognitive impairment (non-dementia)
EQ-5D-5L	N/A	A 5-item questionnaire with a visual analogue scale that evaluates quality of life [51].	N/A
Fatigue Assessment Scale (FAS)	N/A	The Fatigue Assessment Scale is a 10-item scale evaluating mental and physical fatigue symptoms with a total score of 50 [37].	Score 22 or higher indicates fatigue.
Pittsburgh Sleep Quality Index (PSQI)	N/A	The PSQI is a self-report questionnaire that assesses sleep quality in the previous month [38].	Dimensional score 5 or higher indicates poor sleep quality.
WEMWBS	N/A	The Warwick Edinburgh Mental Wellbeing Scale (WEMWBS) (short version) is a 7-item scale which measures multiple aspects of mental wellbeing [39].	Score 42 or lower indicative of poor wellbeing.
Columbia Suicide Severity Rating Scale (C-SSRS)	N/A	The Columbia Suicide Severity Rating Scale (C-SSRS) is a screening instrument for suicidal thoughts and behaviors to identify those at risk of suicide and the severity of that risk [41].	N/A
Short Loneliness Scale (SLS)	N/A	The Short Loneliness Scale (SLS) measures social isolation and is designed for use in large studies [40].	N/A
Change in work status	N/A	A study specific self-report assessment that evaluates change in work status before and after COVID-19 testing.	N/A
