# Supplementary material for: Mental Health and Cognitive Outcomes in Patients Six Months After Testing Positive Compared with Matched Patients Testing Negative for COVID-19 in a Non-Hospitalized Sample: A Matched Retrospective Cohort Study
Source: Int J Environ Res Public Health. 2025 Aug 9;22(8):1249. doi: 10.3390/ijerph22081249 (PMC12386409; doi:10.3390/ijerph22081249)
Supplement: Supplementary file 1 [file ijerph-22-01249-s001.zip › Table S2- Predictors, potential confounders and effect modifiers.pdf]

Table S2. Predictors, potential confounders and effect modifiers

**Predictors and potential confounders**	
Demographic details	Self-reported information on age, ethnicity, gender identity, sexual orientation, education, income, employment, housing and living circumstances
Past mental health history	Self-reported information on previous diagnoses and specialized psychiatric treatment as an outpatient or in-patient.
Current medical problems	Self-reported body mass index and current medical disorders.
Impact of COVID-19 infection	For those who tested positive we will record details of the severity and impact of the COVID-19 infection including neurological symptoms such as anosmia and headaches, length of time self-isolating, time off work, and vaccination status.
Adverse childhood events questionnaire (ACE)	The ACE-Q is a 17 item self-report questionnaire assessing types of adverse exposures during childhood including psychological, physical and sexual abuse as well as household dysfunction. The ACE questionnaire study found strong relationships between childhood exposure and disease conditions, health risk factors and a strong dose-response relationship between number of exposures and risk factors for death [42].
**Effect modifiers**	
Current mental health treatment and outcomes	Self-reported details of current mental health treatment including counselling, psychological treatments and psychiatric medication, as well as self-harm and psychiatric hospitalisation.
Social support	Self-reported social support evaluated by the Oslo Social Support Scale -3 (OSSS-3) a 3-item questionnaire that assesses the level of social support [43].
