# Supplementary material for: Mental Health and Cognitive Outcomes in Patients Six Months After Testing Positive Compared with Matched Patients Testing Negative for COVID-19 in a Non-Hospitalized Sample: A Matched Retrospective Cohort Study
Source: Int J Environ Res Public Health. 2025 Aug 9;22(8):1249. doi: 10.3390/ijerph22081249 (PMC12386409; doi:10.3390/ijerph22081249)
Supplement: Supplementary file 1 [file ijerph-22-01249-s001.zip › Table S3 - Comparison of mean differences in continuous outcomes and regression-adjusted differences.pdf]

Table S3. Comparison of mean differences in continuous outcomes and regression-adjusted differences

	COVID-19 positive		COVID-19 negative		t-test		Regression*			
Continuous variables	Mean	SD	Mean	SD	mean difference	p-value	Beta	95% CI		p-value
PHQ9	5.8	5.3	6.4	5.3	-0.63	0.29	-0.14	-1.24	0.96	0.81
MADRSScore	15.9	8.0	16.8	9.5	-0.88	0.68	-0.40	-4.56	3.77	0.85
GAD7	4.6	4.5	5.5	5.1	-0.86	0.12	-0.40	-1.41	0.60	0.43
HAMA Score	11.7	5.3	14.3	8.6	-2.63	0.19	-0.81	-5.15	3.53	0.71
PCPTSD5	0.5	1.2	0.6	1.3	-0.12	0.39	-0.02	-0.29	0.25	0.87
VAS	75.9	16.0	75.1	16.8	0.79	0.67	0.26	-3.36	3.88	0.89
AUDIT	3.3	3.4	4.8	5.2	-1.50	0.00	-1.28	-2.27	-0.28	0.01
DAST	1.3	0.8	1.5	0.9	-0.12	0.20	-0.12	-0.31	0.06	0.20
FAS	22.2	8.4	22.8	7.6	-0.55	0.54	0.23	-1.43	1.88	0.79
SLS	5.6	2.3	5.9	2.1	-0.31	0.21	-0.16	-0.65	0.32	0.50
PSQI	8.8	3.0	8.4	3.0	0.36	0.29	0.70	0.05	1.35	0.03
Wellbeing	48.8	9.8	48.7	8.8	0.17	0.87	-0.47	-2.43	1.49	0.64
M-ACE	27.0	3.4	27.6	2.4	-0.68	0.05	-0.65	-1.35	0.05	0.07

*Adjusted for age, composite of psychiatric history, and health care worker status

PHQ9: Patient Health Questionnaire; MADRS: Montgomery–Åsberg Depression Rating Scale GAD: General Anxiety Disorder Questionnaire; HAMA: Hamilton Anxiety Rating Scale; PCPTSD: Primary Scare Post Traumatic Stress Disorder Scale; VAS: Visual Analogue Scale; AUDIT: Alcohol Use Disorders Identification Test; DAST: Drug Abuse Screening Test; FAS: Fatigue Assessment Scale; SLS: Short Loneliness Scale; PSQI: Pittsburgh Sleep Quality Index; Wellbeing: Wellbeing scale; M-ACE: The Mini-Addenbrooke's Cognitive Examinations
