# Supplementary material for: Mental Health and Cognitive Outcomes in Patients Six Months After Testing Positive Compared with Matched Patients Testing Negative for COVID-19 in a Non-Hospitalized Sample: A Matched Retrospective Cohort Study
Source: Int J Environ Res Public Health. 2025 Aug 9;22(8):1249. doi: 10.3390/ijerph22081249 (PMC12386409; doi:10.3390/ijerph22081249)
Supplement: Supplementary file 1 [file ijerph-22-01249-s001.zip › Table S4 - Comparison of mean differences in continuous outcomes and regression-adjusted differences- only in participants without psychiatric diagnoses .pdf]

Table S4. Comparison of mean differences in continuous outcomes and regression-adjusted differences- only in participants without psychiatric diagnoses (N=185; 79 COVID-19 negative, 106 COVID-19 positive)

	Cases		Controls		t-test		Regression*			
**Continuous variables**	**Mean**	**SD**	**Mean**	**SD**	**mean difference**	**p-value**	**beta**	**95% CI**		**p-value**
PHQ9	4.4	4.4	5.0	4.3	-0.6	0.39	-0.40	-1.65	0.86	0.53
MADRSScore	12.0	6.6	13.4	6.4	-1.4	0.62	-0.41	-7.36	6.55	0.90
GAD7	3.4	4.1	3.7	3.8	-0.3	0.56	-0.28	-1.45	0.89	0.63
HAMAScore	11.1	5.7	9.8	5.3	1.3	0.61	1.72	-4.29	7.74	0.55
PCPTSD5	0.2	0.8	0.2	0.8	0.0	0.97	0.00	-0.23	0.24	0.97
caps_criteria	5.7	1.5	4.5	3.5	1.2	0.63	1.86	-24.12	27.84	0.53
caps_symptom	8.0	3.5	4.0	5.7	4.0	0.39	5.76	-22.59	34.10	0.24
caps_severity	20.7	8.5	5.0	7.1	15.7	0.12	20.16	9.53	30.79	0.03
VAS	78.2	15.5	78.3	16.7	-0.2	0.94	-0.10	-4.86	4.66	0.97
AUDIT	2.8	2.9	4.2	4.2	-1.4	0.01	-1.47	-2.53	-0.40	0.01
DAST	1.3	0.7	1.4	0.8	-0.1	0.40	-0.10	-0.32	0.12	0.38
FAS	19.9	7.0	20.0	6.4	-0.1	0.94	0.05	-1.93	2.02	0.96
SLS	5.1	2.2	5.5	2.0	-0.4	0.16	-0.37	-1.00	0.25	0.23
PSQI	8.0	2.6	7.7	2.9	0.3	0.41	0.43	-0.39	1.24	0.30
Wellbeing	51.0	9.0	51.0	7.7	0.0	0.97	-0.11	-2.55	2.32	0.93
M-ACE	26.8	3.3	27.7	2.4	-0.8	0.06	-0.87	-1.77	0.02	0.06

PHQ9: Patient Health Questionnaire; MADRS: Montgomery–Åsberg Depression Rating Scale GAD: General Anxiety Disorder Questionnaire; HAMA: Hamilton Anxiety Rating Scale; PCPTSD: Primary Scare Post Traumatic Stress Disorder Scale; VAS: Visual Analogue Scale; AUDIT: Alcohol Use Disorders Identification Test; DAST: Drug Abuse Screening Test; FAS: Fatigue Assessment Scale; SLS: Short Loneliness Scale; PSQI: Pittsburgh Sleep Quality Index; Wellbeing: Wellbeing scale; M-ACE: The Mini-Addenbrooke's Cognitive Examinations
