# Supplementary figures and images for: Mental Health and Cognitive Outcomes in Patients Six Months After Testing Positive Compared with Matched Patients Testing Negative for COVID-19 in a Non-Hospitalized Sample: A Matched Retrospective Cohort Study
Source: Int J Environ Res Public Health. 2025 Aug 9;22(8):1249. doi: 10.3390/ijerph22081249 (PMC12386409; doi:10.3390/ijerph22081249)

**Figure S1. Participant Flow Diagram**

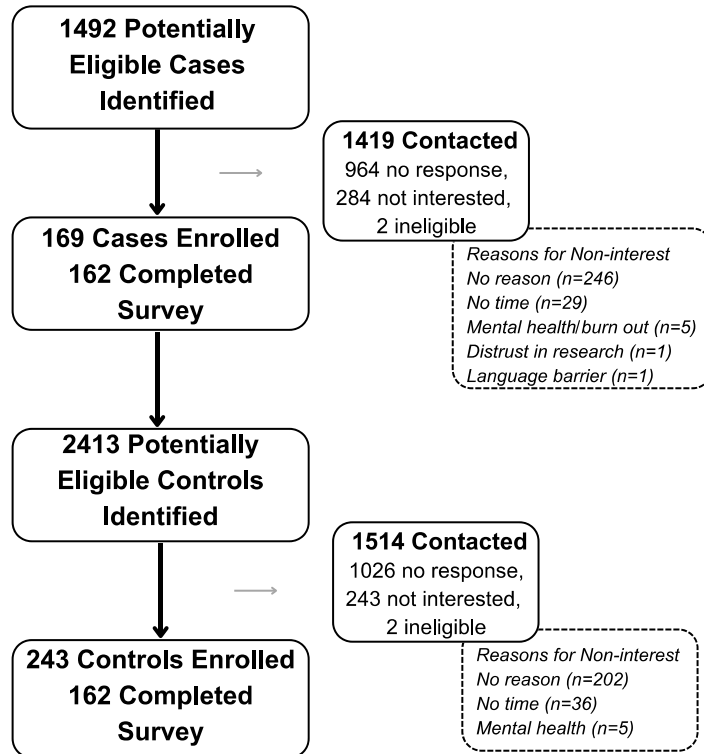

Supplement: Supplementary file 1 [file ijerph-22-01249-s001.zip › Figure S1 - Participant Flow Diagram.pdf]
